# Influence of Silver Fiber Morphology on the Dose–Response Relationship and Enrichment in *Daphnia magna* Studied by Elemental Imaging with LA-ICP-TOF-MS

**DOI:** 10.1021/acs.chemrestox.3c00293

**Published:** 2024-01-08

**Authors:** Tim Steska, Stephan Wagner, Thorsten Reemtsma, Dana Kühnel

**Affiliations:** † Helmholtz Centre for Environmental Research GmbH - UFZ, Permoserstr. 15, 04318 Leipzig, Germany; ‡ Institute for Analytical Chemistry, University of Leipzig, Linnéstr. 3, 04103 Leipzig, Germany

## Abstract

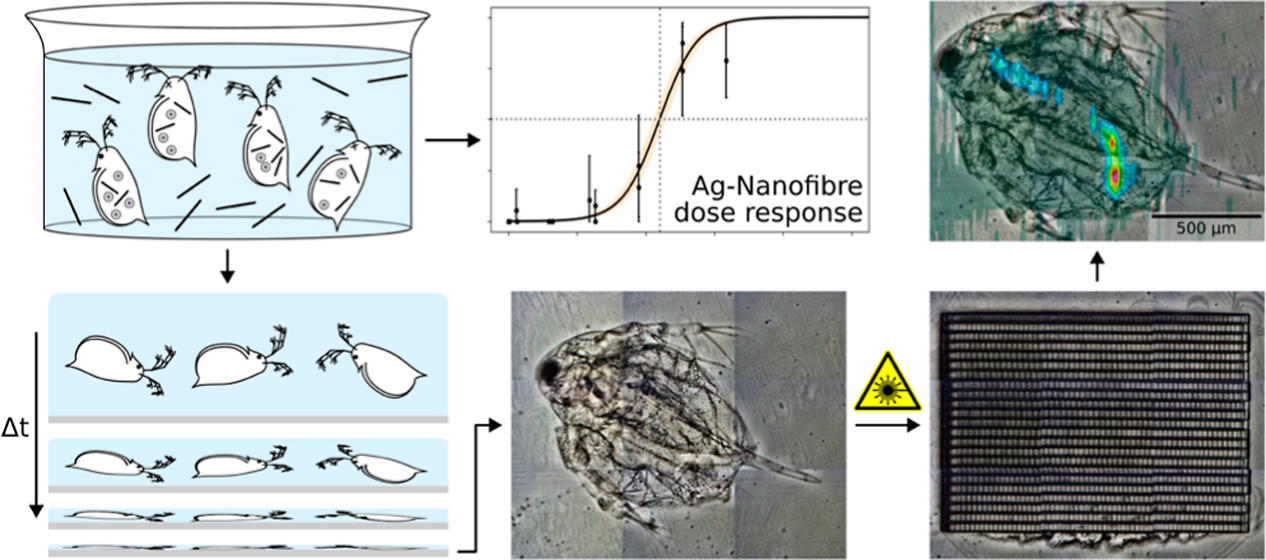

This study aims to enhance the understanding of the environmental risks associated with nanomaterials, particularly nanofibers. Previous research suggested that silver fibers exhibit higher toxicity (EC_50/48h_ 1.6–8.5 μg/L) compared to spherical silver particles (EC_50/48h_ 43 μg/L). To investigate the hypothesis that toxicity is influenced by the morphology and size of nanomaterials, various silver nanofibers with different dimensions (length and diameter) were selected. The study assessed their toxicity toward *Daphnia magna* using the 48 h immobilization assay. The EC_50_ values for the different fibers ranged from 122 to 614 μg/L. Subsequently, the study quantified the uptake and distribution of two representative nanofibers in *D. magna* neonates by employing digestion and imaging mass spectrometry in the form of laser-ablation-ICP-MS. A novel sample preparation method was utilized, allowing the analysis of whole, intact daphnids, which facilitated the localization of silver material and prevented artifacts. The results revealed that, despite the similar ecotoxicity of the silver fibers, the amount of silver associated with the neonates differed by a factor of 2–3. However, both types of nanofibers were primarily found in the gut of the organisms. In conclusion, the findings of this study do not support the expectation that the morphology or size of silver materials affect their toxicity to *D. magna*.

## 1 Introduction

In recent years, nanofibers have gained significant attention, particularly concerning their impact on human health when inhaled. High aspect ratio nanoparticles (HARN) are nanoforms with two similar external dimensions, a significantly larger third dimension, and an aspect ratio of 3:1 or greater.^1,2^ They are commonly referred to as WHO fibers and can deposit in the lungs upon inhalation, impeding effective clearance by macrophages. As a result, inflammatory processes are triggered, and there is a potential to induce cancer.^1−4^

Surprisingly, current environmental risk assessments do not take fiber toxicity into consideration. However, previous research^5−7^ has provided indications that critical fiber dimensions might also exist for aquatic organisms like *Raphidocelis subcapitata* and *Daphnia magna*. Notably, two fibers (Ag – 1340 and SRM110525) and one spherical (NM300 K) silver nanomaterial were investigated, revealing that the fibers exhibit higher toxicity toward *D. magna* (EC_50_ 1.6–8.5 μg/L) compared to the spherical material (EC_50_ 43 μg/L). A higher solubility of the spherical Ag-NM compared to the two fibers suggested that additional factors, such as shape, could influence toxicity. Microscopic images demonstrated an uptake of the fibers by *D. magna* and a presence in the gut.^6,7^

Upon reviewing the current state of knowledge, it became evident that five experimental studies had compared the effects of different silver materials on daphnia, either of different morphologies (e.g., fiber versus nanoparticle) or fibers of different dimensions; in addition, one review was available.^8−13^ Some findings indicated that shorter silver nanowires (10 μm) were more toxic than longer ones (20 μm). In general, fibers were found to be less toxic than silver ions or platelets,^10^ but there were also contradictory results, with longer fibers (20 μm) showing greater toxicity than shorter ones (10 μm).^12^ Another study compared coated silver fibers and found short and SiO_2_-coated fibers to be more toxic to daphnia than longer or PVP-coated fibers.^9^ Additionally, the observed fiber toxicity could not be solely attributed to silver ion release into the media. Comparing silver nanofibers to silver nanoparticles, the latter exhibited higher toxicity in *D. magna* (EC_50_ (fiber) 139 μg/L, EC_50_ (particle) 12 μg/L).^13^ Park et al.^11^ reported an EC_50_ value of 63 μg/L for a silver nanofiber. Consequently, there appears to be a lack of consensus on the influence of different silver nanomaterial shapes and dimensions on toxicity in daphnids, with varying effect values reported in previous studies. Possible reasons for fiber toxicity were speculated to be ion release,^11,13^ uptake of fibers,^9,13^ blockage and damage of the digestive tract,^11,12^ as well as higher energy expenditure due to stress. Further, silver fibers were demonstrated to elicit distinct gene expression profiles depending on size and coating.^9^

The question of whether silver nanomaterial and silver ion uptake and hence internal concentration of silver might be directly correlated to the observed toxicity remains unsolved. This is mainly due to methodological challenges in obtaining quantitative data on total silver internalized by daphnids.^9^ Elemental imaging of biological tissue allows to study of elemental distributions within single organs or in whole organisms.^14,15^ In small model organisms employed in environmental research like zebrafish embryos (ZFE)^16−20^ and daphnids,^17^ the distribution and quantification of chemicals as well as nanomaterials is in particular relevant for studying toxicokinetics.^18,19^ Hence, we employed an LA-ICP-MS approach to study particle morphology effects on uptake into *D. magna* in terms of sufficient resolution and quantification. This will contribute to our understanding of uptake and dose–response relationships of differently-dimensioned silver nanofibers.

It was hypothesized that fiber dimensions (diameter and length) may play a distinct role in toxicity in *D. magna*. As potential mechanisms, a higher uptake, or a reduced excretion depending on fiber length and diameter was assumed, possibly resulting in an increased internal ion concentration, leading to higher toxicity in comparison to the spherical silver particle. Impairment of natural movement and feeding behavior by attachment of fibers to the daphnids was suggested as well.^12,13^

Considering this, our intention in this study was to tackle the following four key hypotheses:

(1) Silver particle morphology influences dose–response curves in *D. magna*, with fiber dimensions (length and diameter) correlating to toxicity in *D. magna*.
(2) Silver particles/fibers represent the main driver of the toxic effect in a dispersion. Dissolved silver, dispersants, and byproducts of the manufacturing process only play a secondary role.
(3) The silver fiber internalization by *D. magna* will determine toxicity; Fiber dimension will determine the amount of internalized silver, and hence toxicity.
(4) Elemental imaging by LA-ICP-MS with an appropriate sample preparation and calibration approach allows mapping of Ag concentrations in *D. magna* in a quantitative manner.

To address the hypotheses concerning fiber-dimension-specific ecotoxicity, we systematically examined seven silver fibers with varying dimensions. For this purpose, we conducted acute 48 h *D. magna* immobilization tests. After determining the EC_50_ values for each fiber, we quantified the amount of internalized silver in *D. magna* neonates exposed to silver fibers at their respective EC_50_ levels. This was done using two methods: digestion of exposed daphnids followed by ICP-MS to determine the total silver associated with the neonates, and laser ablation ICP-MS of whole organisms to assess the distribution of silver within the neonates. To facilitate this process, we developed a novel preparation technique, combining a whole-organism approach (previously demonstrated in ZFE^19^ with a process that is usually used in the preparation of agarose standard slides.^21^ This allowed ablation of whole-body samples without the need for fixation and cutting, minimizing potential artifacts related to silver localization and concentration. The daphnids were embedded in agarose in full and dried, with subsequent laser ablation of the agarose layer including the flattened organisms. This enabled quantification of spatially resolved silver content (based on Böhme et al.^17^ and Halbach et al.^19^) as well as a qualitative differentiation between agglomerated and non-agglomerated silver.

In this study, we present a systematic assessment of silver fiber ecotoxicity and critically discuss each of our initial hypotheses in light of our results, in comparison to previous studies, as well as with regard to methodological challenges.

### 1.1 Experimental Section/Methodology

#### 1.1.1 Silver Fibers and Fiber Dispersion

Five different silver fibers were selected, covering a range of different lengths and diameters, with some fibers having similar lengths but different diameters and vice versa. Four materials were obtained from NANOGAP Europe (A Corua, Spain) and 1 from ACS Material (Pasadena, California, USA). The ACS fiber was further processed by ultrasonic treatment to obtain shorter fibers with equal diameter.^23^ The fiber dimensions as given by the suppliers were re-evaluated by electron microscopy in a previous study^23^ and are shown in Table 1. All materials were delivered as suspensions in an aqueous medium containing an undisclosed quantity of PVP as a dispersant. In the case of Ag_Rod_3170, the suspension had a very high viscosity.

**Table 1:** Overview of Silver Fibers and Their Dimensions as Provided by the Suppliers.^a^

supplier	NANOGAP				ACS materials		RAS^b^	
particle	Ag_Rod_DS_0471	Ag_Rod_3140	Ag_Rod_3143	Ag_Rod_3170	Ag_long	Ag_short	Ag – 1340	SRM110525
length [μm]^c^	1.6	14	1.6	6.5	4.4	1.3	3.8	2.4
diameter [nm]^c^	44.2	52.8	41.1	62.9	52	52	44	241

a RAS fibers used in previous research^6^ are shown for comparison. All fibers were supplied in an aqueous dispersion with PVP dispersant.

b Data from Hund-Rinke et al.^6^

c Previously published in Hund-Rinke et al.^22^

Prior to the preparation of stock dispersions, the supplied fiber dispersions were first agitated on an overhead shaker for 24 h and then vortexed at 220 rpm for 2 min. No ultrasonic treatment was used for dispersal to avoid breakage of fibers. Stock solutions were always freshly prepared on the day of the experiment and at a nominal concentration of 10 mg/L in ADaM (Aachener Daphnien-Medium, according to Klüttgen et al.^24^).

All fiber suspensions were first tested in nominal concentrations of 100 ng/L, 1 μg/L, 10 μg/L, 100 μg/L, 1 mg/L, and 10 mg/L. After the first results indicated the concentration range for EC_50_, suspensions were tested in nominal concentrations of 25, 50, 75, 100, 125, 150, 175, 200, 225, 250, 275, and 300 μg/L with results being corrected to the concentrations determined after the experiment.

#### 1.1.2 Total Silver Concentrations of Exposure Suspensions

To accurately determine total Ag concentrations in ADaM-test suspensions, three replicates of the stock were diluted 1:100 in 60% nitric acid (Ultrapur, Merck KGaA, Darmstadt, Germany) and digested for 2 h at 95 °C and 650 rpm (HLC Cooling ThermoMixer MKR 13, DITABIS AG, Pforzheim, Germany). Concentration was then determined by quadrupole ICP-MS (iCAP RQ ICP-MS, Thermo Scientific, Waltham, MA, USA). For Ag_Rod_3170, these steps were performed before every experiment as the very viscous suspension was very difficult to disperse and dose. For the other materials, it was carried out once.

#### 1.1.3 Fraction of Dissolved Silver in Fiber Dispersions

To differentiate between fiber effects and the effects of dissolved silver ions and dispersant, the liquid phase of two types of fiber dispersion (Ag_Rod_3140 and Ag_Rod_DS_0471) was separated and tested. The preparation of the supernatant samples was performed through centrifugation of two replicates per fiber type in 3 cycles at 11,000 rpm for 25 min with only the supernatant of the previous cycle being transferred to the next. Silver concentration in the supernatant after the final centrifugation step was determined via infusion into time-of-flight ICP-MS (icpTOF R, TOFWERK AG, Thun, Switzerland). By using a dwell time (the time span over which a single point of data is captured) of only 2 ms and monitoring the signal for spikes (as in single-particle ICP-MS), it was confirmed that no fibers remained in the final supernatant.^25^

Supernatant samples were first tested based on nominal particle concentrations of 100 μg/L, 1 mg/L, 10 mg/L, and 100 mg/L, after which the range was refined to 10, 25, 50, 75, and 100 mg/L.

#### 1.1.4 *D. magna* Cultivation

*D. magna* were cultured in mass culture, each flask holding 30 specimens of the same age range in 1.2 L of ADaM. The culture was kept at 20 ± 2 °C under a natural day-night-cycle. The feeding regime was derived from Knops,^26^ feeding thrice weekly with the green algae *Scenedesmus vacuolatus* with an additional supplementation of yeast once a week. Adult survival, number of offspring, and number of ephippia (if present) were tracked to ensure healthy culture conditions (data not shown). Medium replacement and extraction of neonates for testing were performed through staggered sieving.

#### 1.1.5 Miniaturized *D. magna* Acute Immobilization Assay

The 48 h acute toxicity test with *D. magna* (Crustacea, Branchiopoda, Cladocera) was based on OECD TG 202.^27^ Deviating from the OECD guideline, daphnids were exposed in a miniaturized assay in 24-well microplates (TPP, Switzerland), allowing the use of lower volumes of fiber dispersions.^22,28^ Each treatment and control consisted of 4 replicates with 5 daphnids per well (20 animals per test concentration in total), exposed in a total volume of 1.5 mL of ADaM. Neonates were not older than 24 h, and no offspring of adults’ first brood were used. Each test was repeated at least three times, starting the experiments on different days. Fiber suspensions were prepared overconcentrated in order to reach the target concentration after the addition of neonates in 100 μL of ADaM.

Potassium dichromate served as a reference chemical with known toxicological effect values to ensure the constant sensitivity of neonates. During the 48 h of exposure, no food was provided. Immobility was evaluated after 24 and 48 h of exposure by microscopic inspection. The attachment and internalization of silver fibers by daphnids was monitored by light microscopy after the end of the 48 h exposure period and pictures were taken.

The following test validity criteria were applied and met. The immobilization in the negative control was less than 10% (observed: 0%), the pH of the medium ranged between 6 and 9 (observed: 7–7.8), the dissolved oxygen concentration in the medium was ≥3 mg/L (observed: >8 mg/L), and the EC_50/24h_ of reference substance potassium dichromate (K_2_Cr_2_O_7_) ranged between 0.6 and 2.1 mg/L (observed: 1.1 to 1.9 mg/L).

#### 1.1.6 Determination of Silver Uptake by Digestion

After determination of EC_50_ values for all fibers, organisms were exposed to Ag_Rod_3140 and Ag_long at their respective EC_50_ in the Miniaturized *D. magna* acute Immobilization Assay. Following 48 h of exposure, animals were collected and washed three times with ADaM. To ensure that exposure took place during the whole testing duration, only the mobile neonates were collected, while the immobilized fraction was discarded. The remaining organisms were separated into two to three aliquots (each containing 12–108 animals, depending on daily availability), the medium was drained from each, and they were then digested for 2 h at 95 °C and 650 rpm in 2.5 mL of 60% nitric acid. Determination of the total silver content in the digestate was performed using quadrupole ICP-MS. Tests were carried out in duplicate on two different days. In addition, samples of ADaM only and algae feed were digested and analyzed in the same way to determine the background silver concentrations.

#### 1.1.7 Elemental Imaging by Laser Ablation Mass Spectrometry

For calibration purposes, an agarose solution (95 °C, 10 g/L, NEEO Ultra-Qualität, Carl Roth GmbH + Co. KG, Karlsruhe, Germany) was prepared and then spiked with either an indium standard (Merck KGaA, Darmstadt, Germany) or one of two silver fibers (Ag_Rod_3140 and Ag_long). Then, 6.45 mL each were pipetted onto 26 mm × 76 mm glass slides and allowed to cool and dry for several days. Nominal target concentrations were 2 μg/L for indium and 1 mg/L for silver fibers, resulting in 653 pg/cm^2^ and 326 ng/cm^2^, respectively, in the dried layer. To ensure the correctness of the final concentration, samples of silver-spiked agarose were also diluted 1:10 in 60% nitric acid, digested for 2 h at 95 °C and 650 rpm, and then analyzed for Ag using quadrupole ICP-MS. The process is similar to Stärk and Wennrich, 2011,^21^ though no buffer solution is needed for the aqueous silver fiber dispersions.

*D. magna* were exposed, washed, and separated in the same manner as described for the digestion experiments. They were then placed on fresh glass slides (or, in one case, on the previously prepared indium-agarose-coated glass slides) where excess water was removed carefully with a laboratory wipe (as described for ZFE in ref (19)). Again 6.45 mL of agarose solution were pipetted onto the slides and left to cool and dry for several days. The agarose layer reduces in thickness during the drying process, essentially flattening the organism into a 2D projection of its current random orientation. Though depth information is lost in this step, the result is an organism sample that completely lies within the focus plane of the laser, which therefore can be ablated completely in a single pass without the necessity for additional pretreatment, like the laborious preparation of cryo-sections. The depth information lost in this process can be compensated for by the examination of multiple samples of organisms in different orientations.

The dried samples were laser ablated using an excimer laser with a wavelength of 193 nm (Analyte G2, Teledyne CETAC Technologies Inc., Omaha, NE, USA). The laser fluence was adjusted to 5.33 J/cm^2^ pulsing at 125 Hz in a square of 50 by 50 μm while traveling at 25 μm/s. Samples were scanned line by line with an overlap of 10 μm. The resulting theoretical resolution amounts to 0.2 μm in the scan direction and 40 μm perpendicular to the scan direction (or, when rendered as a 2D-plot, 5000 × 25 pixels for a 1 mm × 1 mm square). In a preliminary experiment, the indium-spiked double-layered samples were used to confirm the complete ablation of the target area at these laser settings. A constant indium signal was observed, indicating a complete ablation of the area of interest above. Sampling closer than 6 mm to any edge of the slide was also avoided as the agarose layer does not dry homogeneously near its border.^21^

The laser system was coupled to a TOF-ICP-MS system, measuring with dry plasma and recording signal intensity for ^115^In and ^107^Ag as well as the laser focus position. The performance of the system was calibrated daily using a tuning solution (Inorganic Ventures, Christiansburg, VA, USA) and the prepared indium-spiked standard layer.

For calibration purposes and to compensate for signal drift, before and after the measurement of each specimen, ablation of the corresponding particle-spiked sample was carried out.

### 1.2 Data Evaluation

#### 1.2.1 Miniaturized *D. magna* Acute Immobilization Assay

For each silver fiber and each experiment, the percentage of immobilized neonates after 48 h was plotted against the silver concentration. Respective EC_50_ values and 95%-confidence intervals were derived by fitting a sigmoid curve to the data using a Python script (see Supporting Information).

#### 1.2.2 Laser Ablation Mass Spectrometry

In total, five laser ablation experiments for both Ag_Rod_3140 and Ag_long as well as four unexposed control organisms have been evaluated.

In the first step of producing 2D contour plots, response factors were derived from the measurements taken before and after each sample ablation, which were used to calibrate the sample data in a one-point calibration (Table S2.1). They were taken from agarose samples spiked with a known fiber concentration made from the same fiber present in the sample to accurately compensate for any differences in ablation, fragmentation, transport, nebulization, and ionization behavior. To prevent the onset and trail-off effects from influencing the calibration, the first and last 10% of each ablated line were discarded and an arithmetic mean was calculated from the remaining data. As the sample measurements would often take an hour or more, signal drift had to be accounted for. Assuming a linear drift, calibration was performed with a linear gradient from pre-to postablation measurement, scaling each data point according to their distance to the start/end of the experiment (for additional explanation see Section S2).

The calibrated data were visualized as a 2D contour plot on top of light micrographs using the coordinate information supplied by the laser system. To prevent single-point fluctuations from dominating the scale, a Gaussian filter was applied before plotting. The total silver amount was determined by averaging the calibrated signal over the sample area and multiplying it by said area. All data evaluations were performed using a Python script (see Supporting Information).

## 2 Results and Discussion

### 2.1 Silver Fiber Toxicity in Miniaturized *D. magna* Immobilization Assay

**Figure 1:**
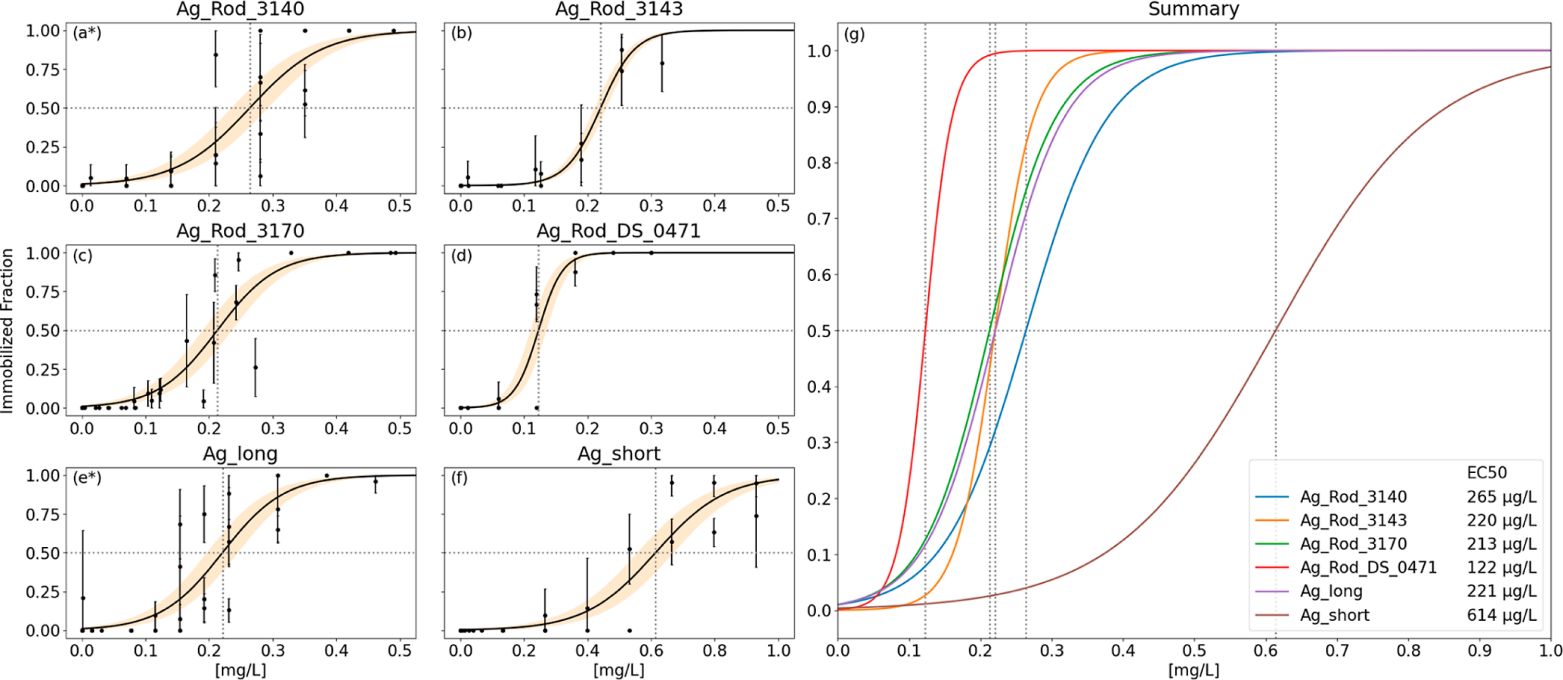
Dose–response relationships of the six silver fibers (a–f) and in comparison (g). Note the different scale for Ag_short (f). Asterisks denote the material used in ablation experiments.

Applying the Miniaturized *D. magna* acute Immobilization Assay, dose–response curves (Figure 1) and EC_50_ values (Table 2) for 48 h of exposure were determined for all fibers. As shown in Figure 1g, Ag_Rod_DS_0471 was the most toxic fiber and Ag_short was the least toxic fiber. In between, the fibers Ag_Rod_3170, Ag_Rod_3143, Ag_long, and Ag_Rod_3140 show almost similar toxicity. EC_50_ values computed from the dose–response curves ranged from 122 μg/L (Ag_Rod_DS_0471) to 265 μg/L (Ag_Rod_3140), leaving a gap to Ag_short at 614 μg/L (Table 2).

**Table 2:** Summary of Dimensions, Share of Dissolved Silver, and *D. magna* Toxicity for the Silver Fibers Studied.^a^

supplier	NANOGAP				ACS materials		RAS^b^	
particle	Ag_Rod_DS_0471	Ag_Rod_3140	Ag_Rod_3143	Ag_Rod_3170	Ag_long	Ag_short	Ag – 1340	SRM110525
length [μm]^c^	1.6	14	1.6	6.5	4.4	1.3	3.8	2.4
diameter [nm]^c^	44.2	52.8	41.1	62.9	52	52	44	241
Ag fraction in solution	0.52%	0.63%	0.25%	0.34%	0.51%	0.08%		
solubility [mg/L]^c^	0.04	0.05	0.04	n.d.	0.03	n.d.	4	0.04
EC_50/48h_ [μg/L]	122 [110–134]	265 [251–279]	220 [212–229]	213 [191–235]	221 [198–243]	614 [549–669]	1.6	8.5
EC_50/48h_ of liquid phase^d^	1/606 (384 μg/L)	1/354 (591 μg/L)						

a For comparison, data for two fibers and one spherical silver particle tested in previous studies^6^ are included.

b Data from Hund-Rinke et al.^6^

c Previously published in Hund-Rinke et al.^22^

d EC_50_ of liquid phase divided by EC_50_ of full suspension. Silver concentration determined in liquid phase at EC_50_ in brackets.

No correlation between fiber morphology and the dose–response of *D. magna* could be established in the assessment. Neither the length nor diameter of the fiber seems to affect the acute response of the test organisms, with EC_50_ values after 48 h between 122 and 265 μg/L. The only exception from this lies in the particle Ag_short at 614 μg/L which was manufactured from Ag_long in the scope of the InnoMat. Life project. Here, the shorter length by retaining the same diameter led to a reduction in toxicity. As this material was not supplied by a professional producer it cannot ensured that all relevant parameters (e.g., surfactants, surface structure, and ion release) remained unchanged.^22^

Compared to the silver fibers from the previous studies, the EC_50_ values for the 5 silver fibers in this study are roughly 3 orders of magnitude higher, and hence the two silver fibers Ag – 1340 and SRM110525 show a higher toxicity.

Based on these results, our first hypothesis, that particle dimensions correlate with toxicity in silver nanowires, can be rejected.

### 2.2 Liquid Phase Toxicity

In almost all suspensions, dissolved silver was responsible for about 0.5% of the total silver concentration (0.25–0.63%) with the only exception being the freshly prepared material Ag_short with 0.08%. The assessment of the supernatants from fiber dispersions of Ag_Rod_DS_0471 and Ag_Rod_3140 in acute immobilization tests showed a decrease in toxicity after 48 h by a factor of 606 and 354 respectively compared to a full fiber suspension (Table 2).

Looking at the fraction of dissolved silver in the supernatants, an EC_50/48h_ of 384 μg/L for Ag_Rod_DS_0471 and an EC_50/48h_ of 591 μg/L for Ag_Rod_3140 can be observed. However, this toxicity is much lower than would be expected from the dissolved silver content.^29^ While this result differs from the observation of some groups who observed liquid phase toxicity close to the level of silver nitrate^30^ it falls in line with other experiments registering little to no toxicity for their particle supernatant.^10,31^ This difference between expected and observed toxicity implies unavailability for the test organisms, likely due to the formation of silver chlorides in the medium as well as complexation.^32^

As the supernatants of the examined fiber dispersions showed no effect on the animals up until a concentration much higher than the one present in the fiber experiments, and because no additional dispersants were added during the experiment, it can be concluded that the fibers themselves represented the main driver behind the toxic effect and hence support our second hypothesis.

### 2.3 Determination of Total Silver Uptake in *D. magna* Neonates after 48 h of Exposure by Digestion-ICP-MS and LA-ICP-MS

The average total amount of silver present in the exposed neonates was determined by two parallel approaches: in digestate by ICP-MS and by LA-TOF-ICP-MS combined with agarose gel calibration. The results obtained by the two approaches agree reasonably well (Table 3, raw data provided in Tables S1.1 and S1.2, Figure S1).

**Table 3:** Summary of the Average Total Silver Accumulation per Neonate Determined by Digestion in 60% Nitric Acid and by Laser Ablation after 48 h of Exposure.^a^

	total Ag per neonate [ng]		
material	digestion	laser ablation	ratio
Ag_Rod_3140	16.6 [10.7–22.4]	26.9 [12.5–41.3]	1.62
Ag_long	49.9 [43.7–56.1]	66.1 [22.9–109.3]	1.32
control (no exposure)	0.146 [0.115–0.177]	0.037 [0.014–0.059]	0.25

a Values in brackets show standard deviation. Digestion samples represent the average of multiple organisms. Sample size digestion/LA: Ag_Rod_3140 *n* = 6/*n* = 5; Ag_long *n* = 5/*n* = 5; control *n* = 3/*n* = 4.

Exposure to the material Ag_Rod_3140 resulted in an average of 17 and 27 ng of silver present per neonate after 48 h, for the digestion and the LA method, respectively. For the exposure to Ag_long, both methods consistently found higher silver accumulation of 50 and 66 ng silver per neonate. Comparing the two approaches, a student’s *t*-test shows the difference between digestion and LA method as not significant (Ag_Rod_3140: *p* = 0.18; Ag_long: *p* = 0.49). It should be noted that the data are not gathered from the same individuals, as both analytical approaches are destructive. Therefore, the deviation between digestion and LA data summarizes differences between biological replicates as well as between the analytical methods. The higher standard deviation obtained for the laser ablation data may indicate a lower precision of this method, integrating over thousands of laser spots for each daphnia. However, it may also reflect the variability in the Ag uptake of the individual organisms. The digestion approach averages over 12–108 individuals that were processed together. While digestion delivers a more robust average value, it does not allow determination of differences between individuals. Control experiments with unexposed daphnia showed very low silver amounts of 0.15 and 0.04 ng per neonate (Table 3).

The two fibers for which internalization in daphnids was quantified showed comparable toxicity with EC_50/48h_ values of 265 and 221 μg/L (Table 4). The amount of internalized silver, however, differed by a factor of about 2–3 (Table 3). The only difference between these particles known to us lies in their morphology, both possessing an average diameter of 52 nm but differing in their average length by a factor of 3.2 (Ag_Rod_3140: 14 μm; Ag_long: 4.4 μm).

**Table 4:** Silver Uptake after 48 h of Exposure at EC_50_ per Neonate and Extrapolation for All Five Neonates in Each Well in Comparison to the Total Silver Present in Each Well.

			uptake per neonate		uptake per 5 neonates	
material	EC_50/48h_ [μg/L]	Ag/well [ng]	digestion (%)	ablation (%)	digestion (%)	ablation (%)
Ag_Rod_3140	265	397.5	4.2	6.8	20.9	33.8
Ag_long	221	331.5	15.0	19.9	75.2	99.7

This indicates that while fiber morphology might not play a role in acute toxicity, it might very well influence uptake into and long-term accumulation inside of the organism. This could also influence effects in chronic assays (typically ≥21 days) as well as point toward increased risk for particles of certain morphologies to enrich further up the food chain. It could also be the case that the fibers differ in their ion release once ingested by the organism, resulting in similar toxicity despite a difference in uptake. As the determined amount of silver includes both silver that has been absorbed into the organism as well as silver that has been merely ingested, whole-body accumulation has been shown to be a poor indicator of toxicity by Glover and Wood.^33^ When compared to the total amount of silver available in the test vessel, it appears that in the case of Ag_long, most of the present silver is internalized (75–100%; Table 4).

Compared to previous experiments where the ablation of organism slices allowed only qualitative observation of uptake,^17,34^ the approach of ablating the daphnid in full allows us to assess the internal dosage quantitatively. For ablation of ZFE in full, Halbach et al.^19^ were able to demonstrate reasonable agreement between digestion and a nonembedded laser ablation approach regarding the determination of bromine content per organism with differences between −9% and +34%.

### 2.4 Silver Localization in *D. magna* Neonates after 48 h of Exposure

Two silver fibers (Ag_Rod_3140 and Ag_long) were selected for the subsequent analysis of silver localization in the test organism by Laser ablation ICP-MS. Despite similar toxicities, diameter, and silver ion release, the length of fibers differed by a factor of about three, with Ag_long 4.4 μm long compared to 14 μm for Ag_3140.

**Figure 2:**
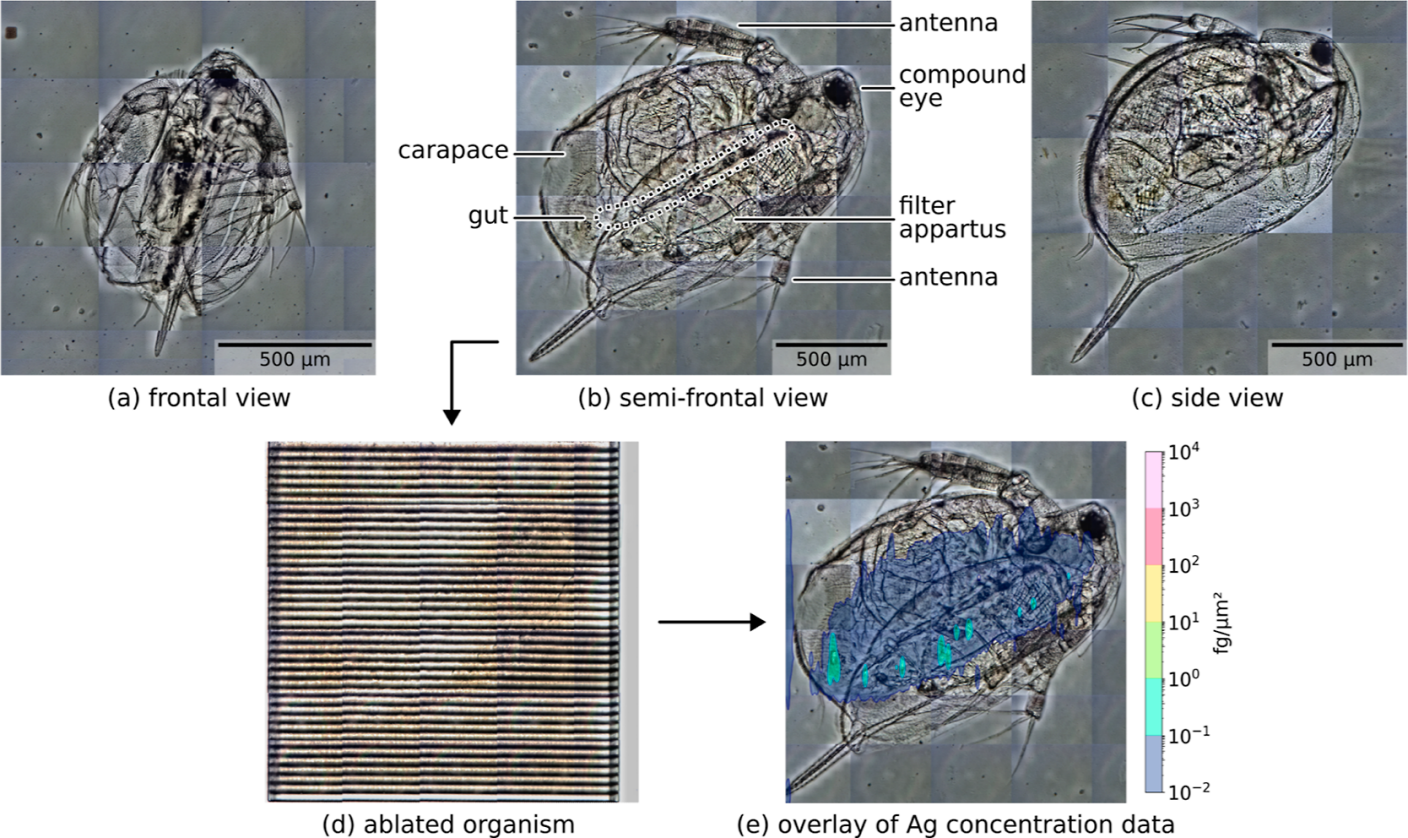
(a–c) Micrographs of different observable orientations of *Daphnia magna* embedded in agarose. Basic elements of Daphnia anatomy are highlighted in (b). (d) Same area as (b) after laser ablation. Mobilized material is measured in real time via TOF-ICP-MS and silver concentration can be overlaid onto the preablation image. (e) Spatially resolved and calibrated silver concentration data for a nonexposed organism from the control group still showing silver presence in the animal’s gut.

In Daphnia, the round carapace and the resulting small contact surface between the slide and the animal usually prevent treatment of the whole organism in destructive experiments, as the sample tends to fragment and detach during the procedure. Hence, to allow the analysis of whole organisms, a novel preparation method was employed, allowing for a unique view into exposed organisms. Due to the flattening occurring during the agarose drying process, the whole organism can be ablated in a single ablation experiment (Figure 2). This approach has several advantages: (a) it omits the cryo-sectioning, which requires laborious sample preparation, is prone to artifacts from fixation, and allows to glimpse in a restricted area or plane only (as used in refs (17) and (34) or with TEM in ref (35)); (b) as the whole organism is ablated, similar to ZFE,^19^ it provides higher sensitivity and a more straightforward quantification. Though depth information is lost in this process, easier preparation allows for more samples to be examined. Daphnids will orient themselves at random during the drying process (Figure 2a–c), enabling us to image them from multiple perspectives. Overlaying the data onto micrographs and looking at multiple samples in different orientations allow the identification of organs coinciding with silver accumulation.

In the future, a single-particle LA-ICP-MS based approach (as used in the treatment of singular organs^9^ and sediment samples^36^ might be possible with careful fine-tuning of the process and would allow for differentiation between different particulate species as well as other forms of silver (e.g., ions, complexes).

The silver concentration of the calibration slides was derived from the silver concentration determined from the particle-spiked agarose gel used in their manufacturing process (e.g., for Ag_Rod_3140 silver fiber concentration in agarose was 1.100 mg/L resulting in an area concentration of 4.582 fg/μm^2^ on the slide). These values, in conjunction with measurements of the calibration slides before and after each specimen, were used to calibrate the LA-ICP-MS data for silver.

**Figure 3:**
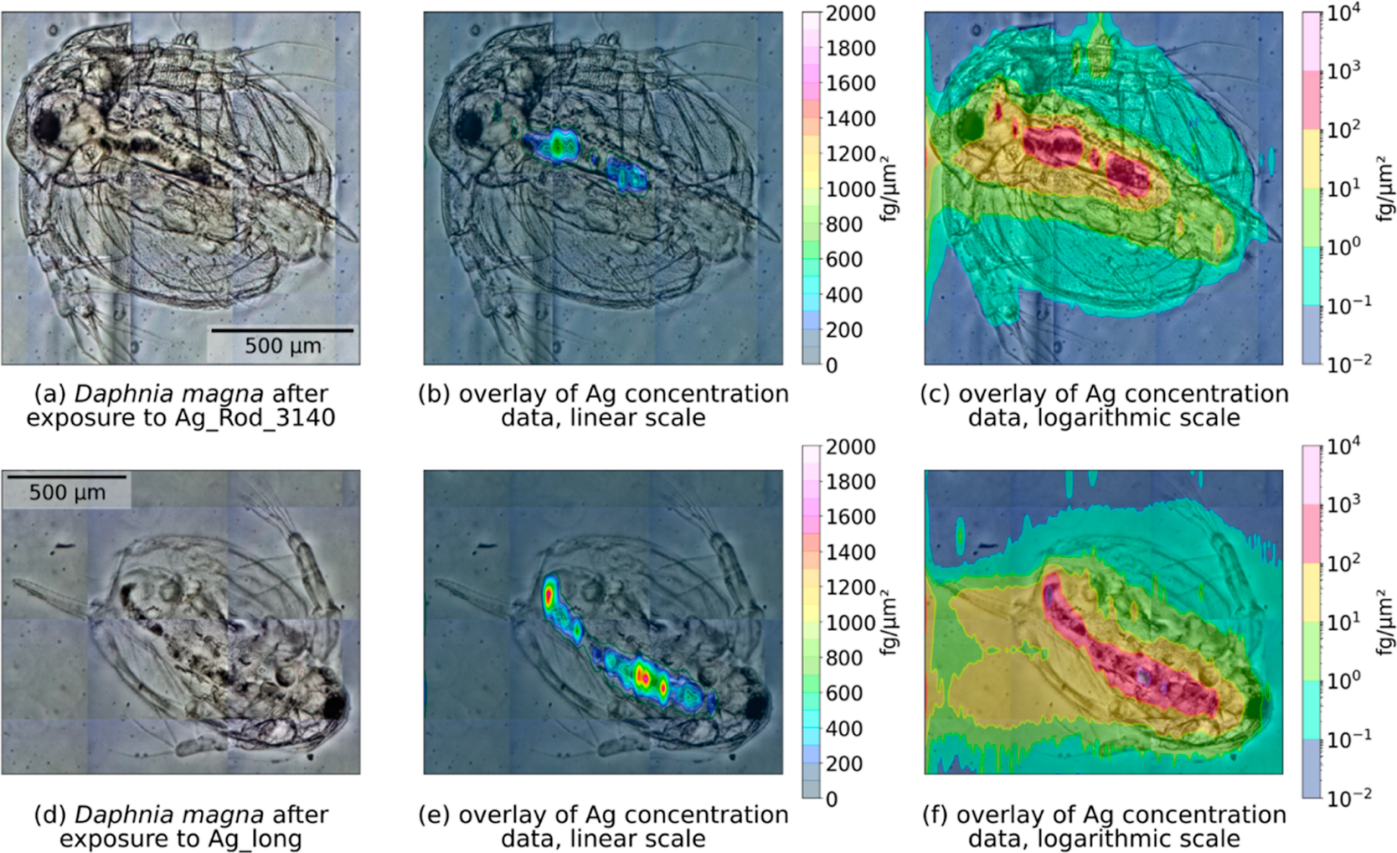
Silver distribution in *Daphnia magna*, visualized by LA-ICP-TOF-MS. (a–c) Specimens were exposed to Ag_Rod_3140, (d–f) specimen exposed to Ag_long at EC_50_ for 48 h and then embedded in agarose. (a,d) Preablation micrograph of the organism; (b,e) overlay of the spatially resolved and calibrated Ag concentration data on a linear scale; (c,f) on a logarithmic scale. High and inhomogeneous silver accumulation can be observed in the daphnid’s gut with a more homogeneous concentration gradient spreading outward from the gut throughout the rest of the organism.

2D-silver-concentration plots revealed silver accumulation in all samples of exposed daphnia. Silver was found primarily in the gut of the animals (Figure 3b,c,e,f). While our LA-ICP-MS approach does not provide sufficient spatial resolution to detect single silver particles, the small steps in which the laser proceeds during measurement allow one to visualize rapid changes in the spatial signal (Figure 3b,e), indicating the presence of fiber agglomerates rather than ionic silver.

Concentration distribution in the rest of the organism appears to follow a smooth gradient from the gut outward and shows a weaker and more homogeneous signal, suggesting uptake of ionic or at the very least nonagglomerated silver fibers into the tissue surrounding the gut. As the animal’s carapace is not directly connected to the circulatory system, a small amount of silver (<1 fg/μm^2^) is likely attached to it during the exposure. These results also concur with previous research where (silver) particles,^17^ copper oxide particles,^35^ polystyrene beads,^37^ and ionic silver^38^ were also found primarily in the gut post-exposure.

These results in combination with the data shown in Table 3 confirm our fourth hypothesis that agarose gel calibration LA-ICP-MS of embedded organisms allows for imaging of Ag concentrations in individual organisms in a quantitative manner. The method’s accuracy, though, is limited by the low number of individuals that can be ablated in a feasible amount of time.

Trace amounts of silver could also be found in the guts of unexposed control neonates (Table 3, Figure 2e), but these were negligible compared to the exposed organisms.

Every 2D plot of a silver-exposed organism shows a slight signal to the left of sample areas containing silver. This is most visible at the very left border of the plot. These signal areas likely are products of the sampling method. During a line scan from left to right, each laser pulse has a 99.6% overlap with the previous pulse, but at the very beginning of the line, there is no previous pulse to overlap with. This leads to a lot of material being mobilized all at once, flushing through the tubing, and taking along previously settled silver. The areas can then be seen as leftover signals of the scan line before. The effect of this developing carryover zone was examined by quantification of silver in only the leftmost 5% of each sample area. It represented only 1.4% [0.8–2.0%] of the total amount in Ag_Rod_3140 and only 2.5% [1.3–3.7%] in Ag_long. We therefore assume that this effect does not significantly impact the quantitative imaging.

Based on these results, our third hypothesis, that fiber internalization will determine toxicity and uptake will depend on fiber dimensions, has to be rejected, although some aspects of it are supported. While the fiber dimension appears to determine the amount of internalized silver, this does not seem to in turn affect toxicity.

## 3 Conclusions

We followed up on the specific toxicity of nanofibers for the aquatic organism *D. magna* by assessing their dose-dependent toxicity and in parallel determining the amount and location of silver that is associated with the organisms by combining and comparing two analytical methods. As a basis for our study, we formulated four distinct hypotheses regarding the relationship between silver fiber properties and potential interactions with *D. magna*. After careful consideration of our results, two of them were supported and two were rejected:

(1) Silver particle morphology influences dose–response curves in *D. magna*, with fiber dimensions (length and diameter) correlating to toxicity in *D. magna*. This hypothesis is rejected, as all fibers showed similar toxicity while varying in length and diameter. The high toxicity of the two silver fibers examined in a previous study^6^ differed substantially from the toxicity of the fibers selected for this study.
(2) Silver particles/fibers represent the main driver of the toxic effect in a dispersion. Dissolved silver, dispersants, and byproducts of the manufacturing process only play a secondary role. This hypothesis was supported by our findings from supernatant testing, indicating a tremendously reduced toxicity upon removal of the silver fibers.
(3) The silver fiber internalization by *D. magna* will determine toxicity; Fiber dimension will determine the amount of internalized silver, and hence toxicity. While certain aspects of this hypothesis are supported, the hypothesis as a whole is rejected. Our findings indicate a correlation between fiber dimension and the amount of internalization, but the extent of silver uptake was not, in turn, correlated with the observed toxicity of the particle suspension.
(4) Elemental imaging by LA-ICP-MS with an appropriate sample preparation and calibration approach allows mapping of Ag concentrations in *D. magna* in a quantitative manner. This hypothesis was supported on the level of an individual organism by our research as the amount of silver determined by laser ablation ICP-MS combined with agarose gel calibration showed significant variability between organisms but, on average, is in agreement with values determined via a digestion approach.

Although extensive reviews on the properties of spherical silver nanoparticles and their impacts on biological systems are available,^39^ to our knowledge this study is the first to systematically investigate the correlation between the morphology of silver nanofibers and the interaction with a model organism. Relationships like these are relevant for grouping approaches as well.^6,40^ While no direct relationship between morphology and acute toxicity could be established, we do hope that this research will serve as a jumping-off point for further studies. Chronic assays examining the long-term effects of a difference in silver uptake and examinations of the role that is played by dispersants or even the manufacturing process of a nanomaterial are needed to give a holistic insight into the interactions between nanofiber and aquatic ecosystems. Background knowledge on fiber synthesis and auxiliary substances in suspensions is generally not disclosed but may play a significant role in regard to short- and long-term toxicity. A more detailed understanding would serve as the basis for read-across^41^ hazard predictions of particles yet to be developed, minimizing future hazard potential.

## Supporting Information Available

Additional tables and figures containing data used for the determination of total silver content and calibration, as well as more information about the calibration process (PDF)
Python script used for the analysis of laser ablation ICP-MS experiments, used for evaluation of standard plates, calibration of the data, mapping, and determination of total silver uptake (TXT)
