# Supplementary material for: Influence of Silver Fiber Morphology on the Dose–Response Relationship and Enrichment in Daphnia magna Studied by Elemental Imaging with LA-ICP-TOF-MS
Source: Chem Res Toxicol. 2024 Jan 8;37(2):292–301. doi: 10.1021/acs.chemrestox.3c00293 (PMC10880099; doi:10.1021/acs.chemrestox.3c00293)
Supplement: Supplementary file 1 — tx3c00293_si_001.pdf [file tx3c00293_si_001.pdf]

Supporting Information

Influence of Ag fiber morphology on dose-response relationship and enrichment in *Daphnia magna* studied by elemental imaging with LA-ICP-TOF-MS

Tim Steska[1]*, Stephan Wagner[1,#], Thorsten Reemtsma[1,2], Dana Kühnel[1]

[1]Helmholtz Centre for Environmental Research GmbH – UFZ, Permoserstr. 15, 04318 Leipzig, Germany

[2]Institute for Analytical Chemistry, University of Leipzig, Linnéstr. 3, 04103 Leipzig, Germany

*Email: tim.steska@ufz.de

Table of Contents

Section S1: Total Silver Content: Accuracy and Precision **S2**

Table S1.1: Average silver content per daphnid determined by laser ablation and by digestion S2

Table S1.2: Silver content per daphnid determined by laser ablation and by digestion, separated by experiment S2

Figure S1: Plot showing average silver content per daphnid as determined by laser ablation and by digestion S3

Section S2: Calibration of Laser Ablation Experiments **S4**

Figure S2.1: Example of laser ablation performed on particle-spiked calibration sample S4

Table S2.1: Average response from pre- and post-measurements of the particle-spiked calibration samples used for one-point calibration of the daphnia ablations S4

Additional explanations regarding the calibration process S4

Section S1: Total Silver Content: Accuracy and Precision

Table S1.1: Average silver content per daphnid determined by laser ablation and by digestion.

	**Laser Ablation**		**Digestion**	
	Average [ng]	Std. Dev	Average [ng]	Std. Dev.
Ag_Rod_3140	**26,908**	14,402	**16,587**	5,863
Ag_long	**66,115**	43,167	**49,890**	6,171
Control	**0,037**	0,022	**0,146**	0,031

Table S1.2: Silver content per daphnid determined by laser ablation and by digestion, separated by experiment. n shows the number of organisms used to measure each data point (whole organism laser ablation, digestion of multiple organisms).

	Ag_Rod_3140 [ng]	n	Ag_long [ng]	n	Control [ng]	n
Laser Ablation	18,243	1	43,842	1	0,021	1
	30,105	1	146,489	1	0,032	1
	9,452	1	71,048	1	0,019	1
	52,233	1	47,824	1	0,074	1
	24,508	1	21,373	1		
Digestion	28,571	35	50,143	35	0,181	18
	16,458	12	42,778	27	0,153	18
	11,458	24	51,389	108	0,106	18
	17,857	14	44,722	108		
	11,429	14	60,417	108		
	13,750	14				

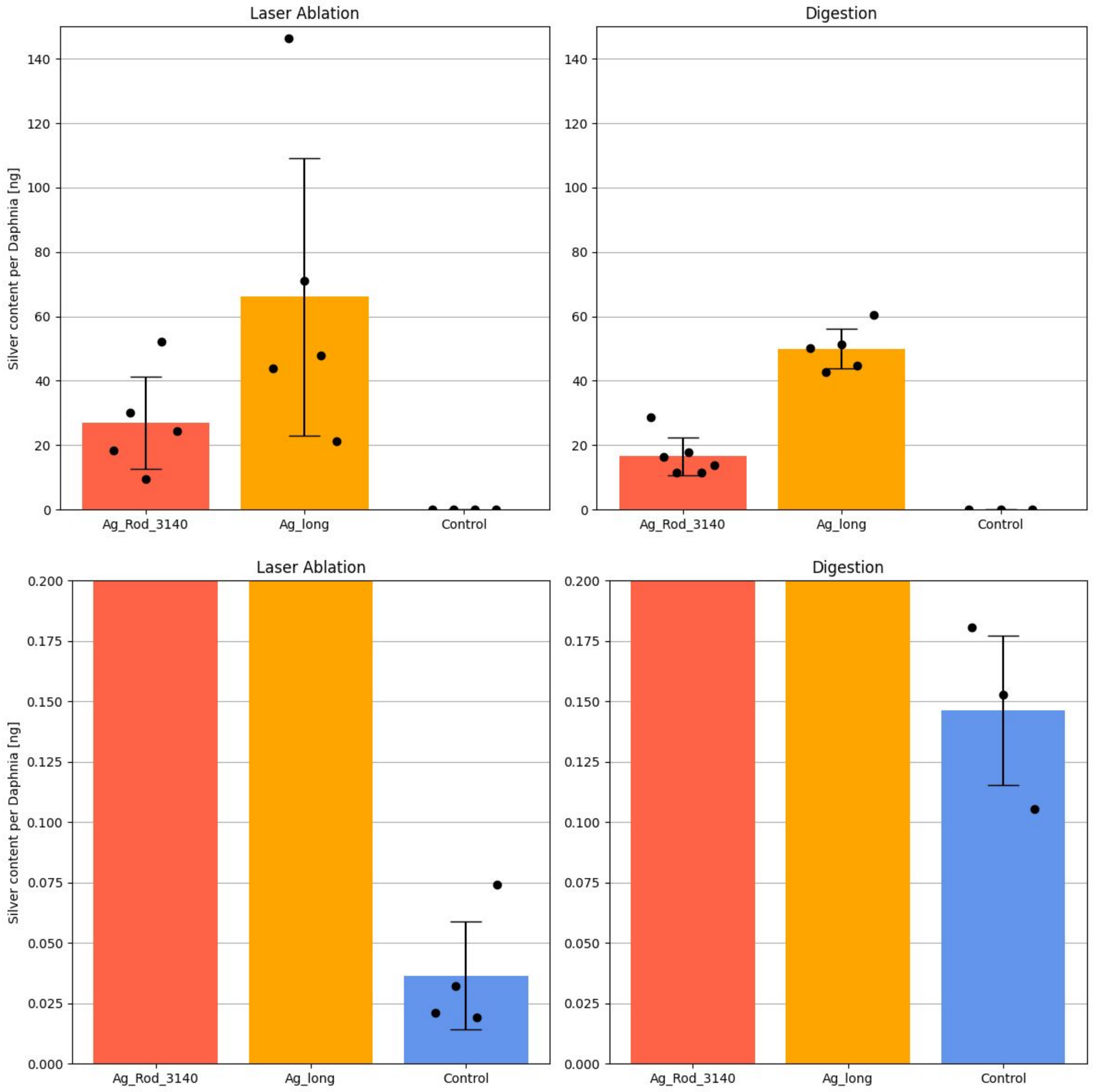

Figure S1: Plot showing average silver content per daphnid as determined by laser ablation (left) and by digestion (right). The average is shown by the height of each bar (Tab. S1.1). Overlaid are individual data (Tab. S1.2) points and standard deviations (Tab. S1.1). Two different scales are used top and bottom to show data for exposed organisms (top) and control (bottom).

Section S2: Calibration of Laser Ablation Experiments

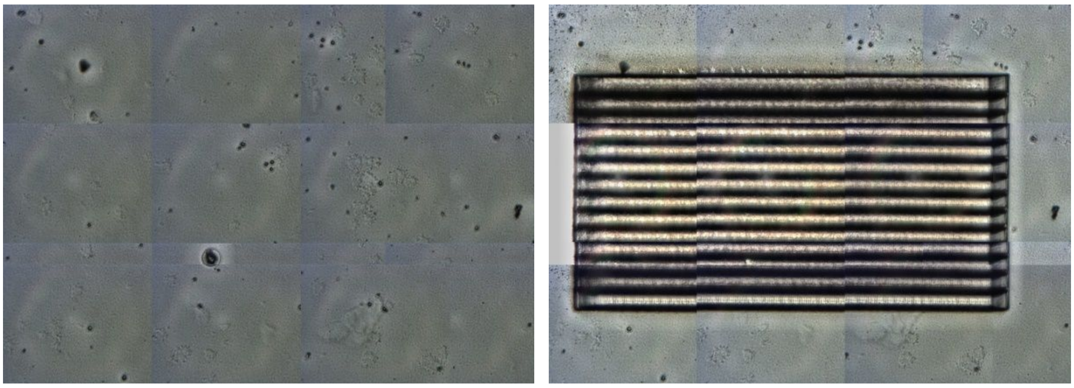

Figure S2.1: Example of laser ablation performed on particle-spiked calibration sample. This was performed before and after each ablation of an organism to compensate for signal drift.

Table S2.1: Average response from pre- and post-measurements of the particle-spiked calibration samples used for one-point calibration of the daphnia ablations. The rightmost column shows the determined silver content per daphnid as shown in Table S1.2 after calibration using the given values was performed.

	pre	post	Ag [ng]
Ag_Rod_3140	1,320	1,274	18,243
	1,313	1,288	30,105
	1,288	1,320	9,452
	0,778	0,607	52,233
	0,607	0,543	24,508
Ag_long	4,920	3,156	43,842
	7,403	7,138	146,489
	3,301	4,920	71,048
	10,660	9,735	47,824
	2,614	2,549	21,373
Control	3,515	3,566	0,021
	3,566	2,143	0,032
	1,213	1,132	0,019
	0,543	0,493	0,074

Due to long measurement times, the following non-classical calibration procedure was applied:
During the preparation of the particle spiked calibration slides, aliquots of the particle spiked agarose solution were diluted 1:10 in 60% nitric acid, digested for 2 h at 95 °C and 650 rpm and then analyzed for Ag using quadrupole ICP-MS. Using the now known silver concentration of the agarose solution, the amount pipetted onto each glass slide (6,45 mL) and the dimension of the glass slides (26 mm × 76 mm), the area concentration of Ag fibers on the calibration slides could be calculated. They were 4.58 fg/µm² and 45.16 fg/µm² for Ag_Rod_3104 and Ag_long respectively. To prevent onset and trail off effects, the first and last 10 % of each ablated line were discarded and an arithmetic mean was calculated from the remaining data.

As the calibration samples were ablated using the same laser setting as the organism samples, the average response factor (signal per fiber and area) of the pre- and post-measurements could be used to calibrate the signal from the organism ablation. To account for signal drift, a linear gradient was calculated from pre- to post-ablation measurement, scaling each data point according to their distance to start/end of the experiment.
